# CircRNA_103809 Suppresses the Proliferation and Metastasis of Breast Cancer Cells by Sponging MicroRNA-532-3p (miR-532-3p)

**DOI:** 10.3389/fgene.2020.00485

**Published:** 2020-05-15

**Authors:** Minfeng Liu, Can Luo, Jianyu Dong, Jingyun Guo, Qing Luo, Changsheng Ye, Zhaoze Guo

**Affiliations:** ^1^Department of Breast, Nanfang Hospital, Southern Medical University, Guangzhou, China; ^2^The First Clinical Medical College, Southern Medical University, Guangzhou, China

**Keywords:** circRNA_103809, miR-532-3p, breast cancer, proliferation, metastasis

## Abstract

Breast cancer has become one of the most serious disease threatening mankind health in the world. Accumulating studies indicated that circRNAs played an important role in the occurrence and progression of breast cancer, however, the roles of circRNA_103809 in breast cancer progression remain unclear. Therefore, in this study, we aimed to clarify the potential role and regulatory mechanism of circRNA_103809 in the development of breast cancer. Firstly, the expression level of circRNA_103809 and microRNA-532-3p (miR-532-3p) in breast cancer tissues and normal tissues were detected with the quantitative real-time polymerase chain reaction (RT-qPCR). In addition, the cell proliferation ability, metastasis ability and related pathways were identified by Cell Counting Kit-8 (CCK-8), flow cytometry, and western blot, respectively. Furthermore, the connection between circRNA_103809 and miR-532-3p was detected by dual-luciferase reporter assay. Then, our data showed that circRNA_103809 was down-regulated in breast cancer tissues in contrast to adjacent non-tumor tissues, and the relative expression level of circRNA_103809 was closely associated with distant metastasis size, TNM stage, HER-2 status and overall survival time. In addition, our *in vitro* assays showed that the overexpression of circRNA_103809 could significantly inhibit epithelial-mesenchymal transition (EMT) pathway, then suppress breast cancer cell proliferation and metastasis ability. Moreover, we also found that the antitumor effect induced by circRNA_103809 could be reversed with the addition of miR-532-3p mimics. Taken together, this study showed that circRNA_103809 could inhibit cell proliferation and metastasis in breast cancer by sponging miR-532-3p, and circRNA_103809 might be a prospective target of breast cancer therapy.

## Introduction

Breast cancer is one of the most common malignancies in the world. Approximately 1,400,000 cases of breast cancer are diagnosed, and 500,000 women die from breast cancer each year (Torre et al., 2015). As a matter of fact, most of the patients were diagnosed as advanced breast cancer, who are suffering from relatively high morbidity rate and high mortality rate (Bray et al., 2018). In recent years, the diagnostic and therapeutic methods have improved greatly (Bachelot et al., 2019; Freedman et al., 2019), but the death rate from adenocarcinoma is still high (Eldredge-Hindy et al., 2014). Therefore, it is of great important to explore novel biomarkers for breast cancer.

Recently, increasing studies have identified that circular RNAs (circRNAs) plays significant regulatory roles in the development of malignant tumors (Ye et al., 2019). In fact, circRNAs, unlike lncRNAs and miRNAs which have 5′-3′ polarity and polyadenylated tails, have closed loop structures (Memczak et al., 2013). Consequently, due to their highly stable structure, circRNAs have very stable biological functions (Qiao et al., 2018). Accumulating studies have elucidated diverse physiological and pathological roles of circRNAs, especially in the generation and development of tumors (Mercer et al., 2011; Hentze and Preiss, 2013). For example, circTADA2As exerts anti-carcinogenic function by sponging miR-203a-3p in breast cancer (Xu J. Z. et al., 2019). Another study demonstrates that circ_0072309 mediates breast cancer cells progression via sponging miR-492 (Yan et al., 2019). However, the biological role of circRNAs in breast cancer progression has not been totally elucidated.

More importantly, circRNA_103809 (circRNA_103809) is a non-coding RNA that located at chromosome 5p13.3 and consisted of 5 exons of ZFR gene. Recently, emerging studies identified that circRNA_103809 played an important role in the development of various cancers, including colorectal cancer (Bian et al., 2018), lung cancer (Liu et al., 2018), and hepatocellular carcinoma (Zhan et al., 2020). For example, Zhan’s research proved that circRNA_103809 participated in the regulation of biological function in hepatocellular carcinoma via regulating miR-377-3p/FGFR1/EPR axis (Zhan et al., 2020). In addition, Bian et al. discovered that circRNA_103809 could regulate the cell metastasis and proliferation abilities in colorectal cancer through sponging miR-532-3p (Bian et al., 2018). However, it was still not clear whether circRNA_103809 acted as an oncogene or tumor suppressor in breast cancer.

In this study, we firstly evaluate the expression level of circRNA_103809 in breast cancer tissue and normal tissue samples, respectively, and then investigate the relationship between the expression of circRNA_103809 and the clinicopathological features of breast cancer patients. Subsequently, we performed *in vitro* experiments to further confirm the biological role and potential mechanism of circRNA_103809 in breast cancer. Moreover, based on the results of bioinformatic analysis, we hypothesized that miR-532-3p might be the downstream gene of circRNA_103809 based. Above all, our data demonstrated that circRNA_103809 might be a promising treatment target for breast cancer.

## Materials and Methods

### Tissue Samples

Breast cancer and paired non-tumor tissues of 65 breast cancer cases were collected at Nanfang Hospital, and the patients had not been previously treated with radio- or chemotherapy. The inclusion criteria were as follows: (1) Age older than 18 and younger than 80 years; (2) Written informed consent; and (3) Primary ovarian cancer confirmed pathologically by experienced pathologists. In addition, the exclusion criteria were as follows: (1) Patients with other malignant diseases and (2) Patients with previous neoadjuvant chemotherapy or radiotherapy. All of the tissue samples was evaluated by experienced pathologists at Nanfang Hospital. Furthermore, this research was approved by the Medical Ethics Committee at our center, and all of the included patients signed informed consent voluntarily.

### Cell Culture

The human breast cancer cell lines (MDA-MB-157, MCF-7, MDA-MB-231, MDA-MB-468, T47D, BT20) and normal breast epithelial cell line (MCF-10A) were purchased from ATCC (Shanghai, China), and cultured with RPMI-1640 (Gibco) containing 1% penicillin/streptomycin (Gibco) and 10% FBS (HyClone, Logan, UT, United States).

### RNA Transfection

Lentiviruses [multiplicity of infection (MOI) of 30] containing pcDNA3.1 plasmids were used to upregulate the expression of circRNA_103809 in two breast cancer cell lines. Then, the treated cells including the circRNA_103809-overexpressing group (OE-circRNA_103809) and the negative control group (OE-vector) were also transfected with a negative control sequence (miR-532-3p NC) and miR-532-3p mimics via Lipofectamine 2000 Transfection Reagent (Thermo Fisher, United States). Target sequences are shown in Table 1.

**TABLE 1:** Sequences of oligomers and primers used in the present research.

Name	Sequence (5′-3′)
CircRNA_103809 forward	ACG CAT TCT TCG AGA CCT CT
CircRNA_103809 reverse	TGC CTG TAA CTC CTC TTC AGT
miR-532-3p forward	CCC TCC CAC ACC CAA GG
miR-532-3p reverse	CCC AGT AGT CGT TCA GTC CA
β-actin forward	CGC TCT CTG CTC CTG TTC
β-actin reverse	ATC CGT TGA CTC CGA CCT TCA C

### Real-Time Quantitative Polymerase Chain Reaction (RT-qPCR)

Total RNA of the specimens and cell lines were obtained by RNAiso Plus reagent (TaKaRa, Tokyo, Japan). Then, RNA was reverse transcribed into cDNA by PrimeScript RT Master Mix (TaKaRa). Finally, the expression levels of circRNA_103809 or miR-532-3p were determined using SYBR Premix Ex Taq II Kit (TaKaRa). The 2^–Δ^ ^Δ^ ^*Ct*^ method (Khare et al., 2019) was applied to detect fold changes. Primer sequences are listed in Table 1.

### Cell Counting Kit-8 (CCK-8) Assay

The treated cells were plated in 96-well plates. After incubation, Cell Counting Kit-8 (CCK-8) (Dojindo, Kumamoto, Japan) was added to the wells at 0, 24, 48, 72, and 96 h and cell viabilities were further detected according to the OD value.

### Flow Cytometry Assay

The effect of circRNA_103809 or miR-532-3p on cell apoptosis and the cell cycle were detected by Annexin V-FITC/PI apoptosis detection kit (KeyGen, Nanjing, China) and cell cycle detection kit (KeyGen), respectively.

### Cell Migration and Invasion Assay

The cell migration or invasion ability were evaluated with the Transwell chambers (Corning, NY, United States) with or without Matrigel, respectively. Treated cells were cultured in 200 μl DMEM with 2% FBS in the upper chambers, while 500 μl DMEM with 8% FBS was supplied in the lower chambers. Twenty-four hours later, the cells were fixed for imaging.

### Prediction of Downstream Molecules Regulated by CircRNA_103809

A publicly available bioinformatic algorithm (Starbase 2.0) was utilized to predict the downstream microRNAs of circRNA_103809.

### Dual-Luciferase Reporter Assay

293T cells were transfected with pmirGLO-circRNA_103809 or pmirGLO-circRNA_103809-MUT plasmid in the presence of miR-532-3p mimics or a negative control (NC). Then, passive lysis buffer (Yubo, Shanghai, China) was applied to lyse the cells, and the relative luciferase activities were monitored by a luciferase reporter assay system (Promega, Madison, WI, United States).

### Western Blot Analysis

After cell lysis and protein extraction, equivalent amounts of protein (30 μg) were separated in a 10% sodium dodecyl sulfate-polyacrylamide gel electrophoresis (SDS-PAGE) gel, transferred to polyvinylidene fluoride (PVDF) membranes. After incubated with 10% BSA, the specific primary and secondary antibodies. Finally, a chemiluminescent system was used for protein visualization.

### Statistics

All data are shown as the mean ± standard deviation (SD). In addition, the data were analyzed with the Student’s *t*-test, Pearson’s test and chi-square test using BM SPSS 20.0. All experiments were performed three times. *P* < 0.05 were considered as significant difference.

## Results

### Up-Regulation of CircRNA_103809 Is Related to a Better Clinical Outcome of Breast Cancer Patients

**FIGURE 1:**
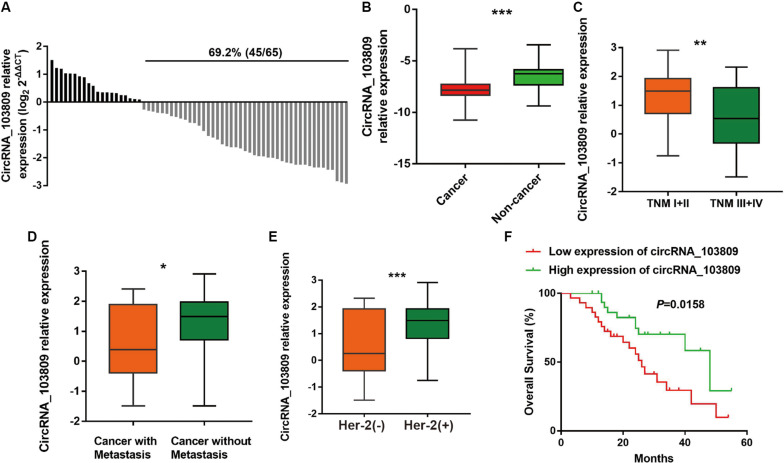
The expression of circRNA_103809 in breast cancer patients and clinicopathological parameters of patients. **(A)** CircRNA_103809 is significantly downregulated (69.2%, 20/65) in patients with breast cancer. **(B)** The expression level of circRNA_103809 is significantly lower in breast cancer tissues than in normal tissues. **(C)** The correlation between the expression of circRNA_103809 and TNM stages. **(D)** The relationship between circRNA_103809 expression and tumor metastasis. **(E)** The relationship between circRNA_103809 expression and Her-2 positive expression. **(F)** The correlation between the expression of circRNA_103809 and overall survival rates of patients with breast cancer. All experiments were repeated at least three times, *P < 0.05, **P < 0.01, ***P < 0.001.

To detect the connection between circRNA_103809 expression and the clinicopathological characteristics of breast cancer patients, RT-qPCR was performed and the results indicated that low expression of circRNA_103809 was detected in 69.2% (45/65) of breast cancer specimens (Figure 1A). In addition, the expression level of circRNA_103809 was down-regulated in breast cancer in contrast to adjacent normal specimens (Figure 1B). Next, according to the median value of circRNA_103809 expression, the patients with gastric cancer were divided into two groups, including high expression group and low expression group. The data showed that the circRNA_103809 expression was closely correlative to the distant metastasis (*P* = 0.045), HER-2 status (*P* < 0.000), and TNM stage (*P* = 0.018) (Table 2). Consistent with these results, breast cancer patients with circRNA_103809 low-expression were more likely to have advanced TNM stage, cancer metastasis and HER-2 negative (Figures 1C–E). Furthermore, the results of Kaplan-Meier (KM) curve revealed that the breast cancer patients with low expression of circRNA_103809 were more likely to have shorter overall survival compared with those with circRNA_103809 overexpression (Figure 1F). Taken together, circRNA_103809 might be considered as a cancer suppressor and even a prospective biomarker for breast cancer.

**TABLE 2:** Clinicopathological variables and the expression of circRNA_103809 in breast cancer patients.

Variable	All cases	CircRNA_103809		*P* value
		Low expression	High expression	
Age (years)				0.724
≤60	25	12	13	
>60	40	21	19	
Tumor size (cm)				0.663
≤2	22	12	10	
>2	43	21	22	
Distant metastasis				0.045
Present	22	15	7	
Absent	43	18	25	
TNM stage				0.018
I-II	35	13	22	
III-IV	30	20	10	
HER-2 status				0.000
Positive	27	23	4	
Negative	38	10	28	
PR status				0.379
Positive	30	17	13	
Negative	35	16	19	
ER status				0.875
Positive	25	13	12	
Negative	40	20	20	

### Overexpression of CircRNA_103809 Inhibits Breast Cancer Cell Proliferation Ability

**FIGURE 2:**
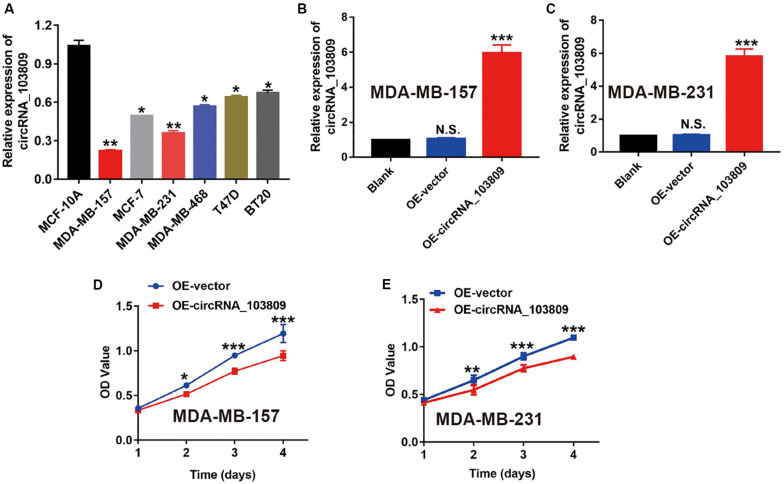
Upregulation of circRNA_103809 suppresses the proliferation of breast cancer cells *in vitro*. **(A)** The expression of circRNA_103809 is downregulated in breast cancer cell lines (MDA-MB-157, MDA-MB-157, MDA-MB-213, MDA-MB-468, T47D, and BT20) compared with a normal breast cell line (MCF-10A). **(B)** Successful generation of circRNA_103809-overexpressing MDA-MB-157 cells induced by plasmid interference. **(C)** Successful generation of circRNA_103809-overexpressing MDA-MB-213 cells induced by plasmid interference. **(D,E)** The effect of upregulation of circRNA_103809 on the proliferation of breast cancer cells *in vitro* measured using a CCK-8 assay. All experiments were repeated at least three times, *P < 0.05, **P < 0.01, ***P < 0.001.

To identify the regulatory role of circRNA_103809 in breast cancer cells, we first detected circRNA_103809 expression in normal MCF-10A cells and breast cancer cells. Then, the RT-qPCR results showed that the expression level of circRNA_103809 in breast cancer cells was dramatically down-regulated than that in normal MCF-10A cells (Figure 2A). Furthermore, MDA-MB-231 and MDA-MB-157 cells were chosen for *in vitro* experiments as the lowest expression of circRNA_103809 was observed in these two cells. We constructed two model cell lines in which circRNA_103809 was overexpressed using plasmid transfection. The results of RT-qPCR indicated that in contrast with the blank (un-transfected) group, the endogenous expression of circRNA_103809 was not changed in the negative control group (OE-vector), while circRNA_103809 levels increased approximately six-fold in the OE-circRNA_103809 group (Figures 2B,C). Subsequently, the OE-circRNA_103809 breast cancer cells were further assayed for *in vitro* cell proliferation ability, and the results of CCK-8 indicated that upregulation of circRNA_103809 could effectively suppress the growth of MDA-MB-157 and MDA-MB-231 cells (Figures 2D,E).

### Upregulation of CircRNA_103809 Arrests More Cells at the G2/M Stage

**FIGURE 3:**
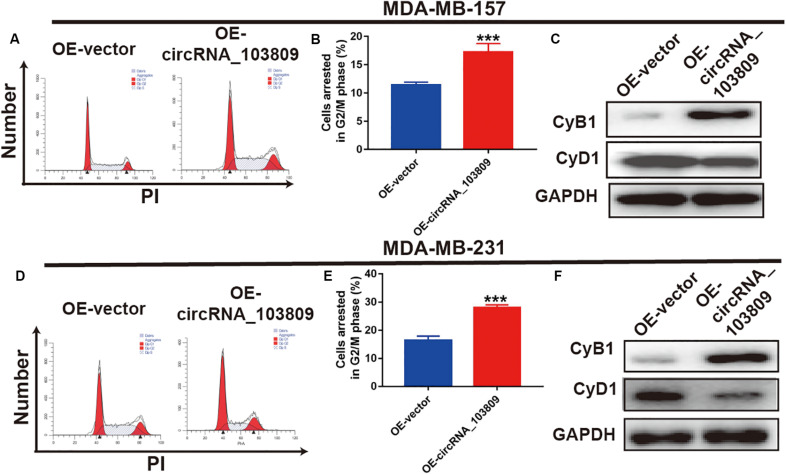
Upregulation of circRNA_103809 arrests breast cancer cells at the G2/M phase and regulates the cell cycle-related pathway in MDA-MB-157, G-63 and MDA-MB-213 cells. **(A,B)** Cell cycle images (left) and flow cytometry statistical analysis (right) of MDA-MB-157 cells after the overexpression of circRNA_103809 in these cells. **(C)** The expression of cell cycle-related proteins (CyB1 and CyD1) in MDA-MB-157 cells detected by western blot analysis after the overexpression of circRNA_103809. **(D,E)** Cell cycle images (left) and flow cytometry statistical analysis (right) of MDA-MB-213 cells after overexpression of circRNA_103809 in these cells. **(F)** The expression of cell cycle-related proteins (CyB1 and CyD1) in MDA-MB-213 cells detected by western blot analysis after the overexpression of circRNA_103809. All experiments were repeated at least three times. ***P < 0.001.

To further clarify the potential regulatory mechanism of circRNA_103809 in breast cancer, we further detected the function of circRNA_103809 on the MDA-MB-157 and MDA-MB-231 cell cycle. As shown in Figures 3A,B, the arrest of more G2/M MDA-MB-157 cells were induced in the OE-circRNA_10380 group (17.2%) than in the OE-vector group (11.1%). In addition, we applied western blotting assay to detect cell cycle-related proteins, and the results showed that the CyB1 expression level was upregulated, while CyD1 expression was decreased (Figure 3C). Similarly, the upregulation of circRNA_103809 could also arrest more MDA-MB-231 cells at the G2/M stage (28.0%) relative to OE-vector group (16.4%) (Figures 3D–F). Accordingly, quantification of western blotting data also showed an increase in CyB1 and a decrease in CyD1 (Supplementary Material and Supplementary Figures S1A,B). In addition, cell apoptosis was evaluated in breast cancer cells. Upon circRNA_103809 overexpression, no significant changes in apoptosis were observed based on the flow cytometry results (Supplementary Material and Supplementary Figure S2). These results suggested that the upregulation of circRNA_103809, might inhibit breast cancer cell growth via arresting more cell at G2/M stage.

### Upregulation of CircRNA_103809 Suppresses Cells Migration and Invasion Ability via Inhibiting Epithelial Mesenchymal Transition (EMT) Pathway in Breast Cancer

**FIGURE 4:**
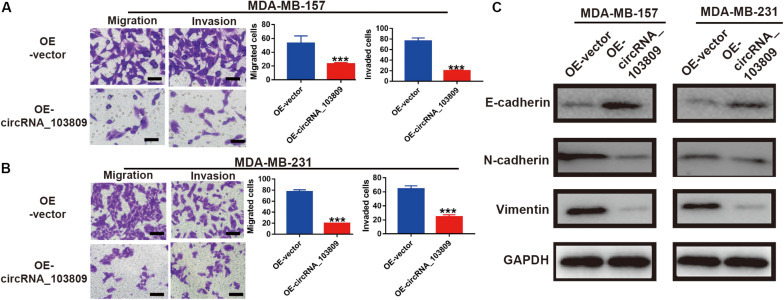
Upregulation of circRNA_103809 inhibits the migration and invasion abilities and regulates the epithelial mesenchymal transition (EMT) process in breast cancer cells. **(A)** Cell migration and invasion images (left) and statistical analysis (right) of migrated and invaded MDA-MB-157 cells after the overexpression of circRNA_103809. **(B)** Cell migration and invasion images (left) and statistical analysis (right) of migrated and invaded MDA-MB-213 cells after the overexpression of circRNA_103809. **(C)** The expression of an epithelial cell marker (E-cadherin) and mesenchymal markers (N-cadherin and vimentin) in MDA-MB-157 and MDA-MB-213 cells detected by western blotting after the overexpression of circRNA_103809. All experiments were repeated at least three times. ***P < 0.001.

To further confirmed the biological function of circRNA_103809 on breast cancer cells, the Transwell assay was carried out to examine the abilities of cell metastasis. When compared with the OE-vector group, the numbers of cell migration and invasion were decreased by ∼57% and ∼74%, respectively after upregulating circRNA_103809 expression in MDA-MB-157 cells (Figure 4A). Consistent with these findings, the numbers of cell migration and invasion were decreased by ∼75.3% and ∼62.5%, respectively after upregulating circRNA_103809 expression in MDA-MB-231 cells (Figure 4B).

To explore the potential anti-metastasis mechanism of circRNA_103809, western blotting was performed and the results indicated that after up-regulation of circRNA_103809, the epithelial marker E-cadherin expression increased significantly, but the mesenchymal markers N-cadherin and vimentin expression decreased dramatically in both MDA-MB-157 and MDA-MB-231 cells (Figure 4C). Accordingly, protein quantification bands also showed same tendency, with epithelial markers increased and mesenchymal markers decreased (Supplementary Material and Supplementary Figures S3A,B). Therefore, these results indicated that circRNA_103809 suppressed cell metastasis through the regulation of EMT in breast cancer.

### MiR-532-3p Is a Direct Target of circRNA_103809 in Breast Cancer

**FIGURE 5:**
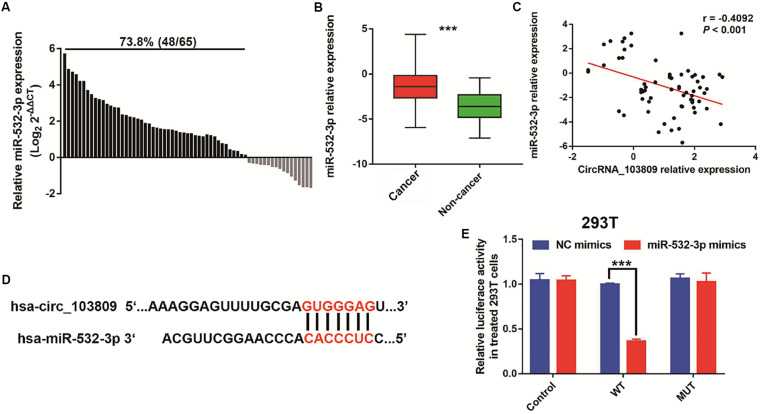
MiR-532-3p is a direct target of circRNA_103809 in breast cancer. **(A)** The relative miR-532-3p expression was upregulated in 73.8% (48/65) of patients with breast cancer. **(B)** miR-532-3p was significantly overexpressed in breast cancer tissues compared with adjacent normal tissues as evaluated by RT-qPCR. **(C)** Correlation between miR-532-3p and circRNA_103809 in paired human breast cancer tissues (*r* = –0.4092, *P* < 0.001). **(D)** The predicted 3′UTR binding regions of circRNA_103809 on miR-532-3p. **(E)** Relative luciferase activity in 293T cells after cotransfection with pmirGLO circRNA_103809-WT or pmirGLO- circRNA_103809-MUT along with miR-532-3p mimics or miR-532-3p NC. ***P < 0.001.

To investigate the downstream targets of circRNA_103809, we attempted to predict its regulatory microRNAs using a public database and identified miR-532-3p as a potential target. To address the role of miR-532-3p in breast cancer, RT-qPCR was carried out and the results indicated that high expression of miR-532-3p was found in 73.8% (48/65) of breast cancer specimens (Figure 5A). Subsequently, the miR-532-3p expression was remarkably upregulated in breast cancer (Figure 5B). Then, to analyze the connection between the expression of circRNA_103809 and miR-532-3p in breast cancer patients, we performed a Pearson correlation study, and we found that these two molecules were negatively correlated at the expression level (Figure 5C, *r* = −0.4092, *P* < 0.001). To validate their association, we predicted the possible binding sites shown in Figure 5D. Next, we constructed wild type (WT)- and mutant type (MUT)-expressing 293T cells using pmirGLO-circRNA_103809-WT or pmirGLO-circRNA_103809-MUT plasmid. A luciferase reporter assay revealed that the luciferase activity was remarkably lower in wild-type-expressing cells transfected with miR-532-3p mimics (Figure 5E). These results demonstrated that miR-532-3p might be the downstream gene of circRNA_103809 in breast cancer.

### MiR-532-3p Mimics Reverse the Antiproliferative Effect Induced by the Overexpression of CircRNA_103809 in Breast Cancer

**FIGURE 6:**
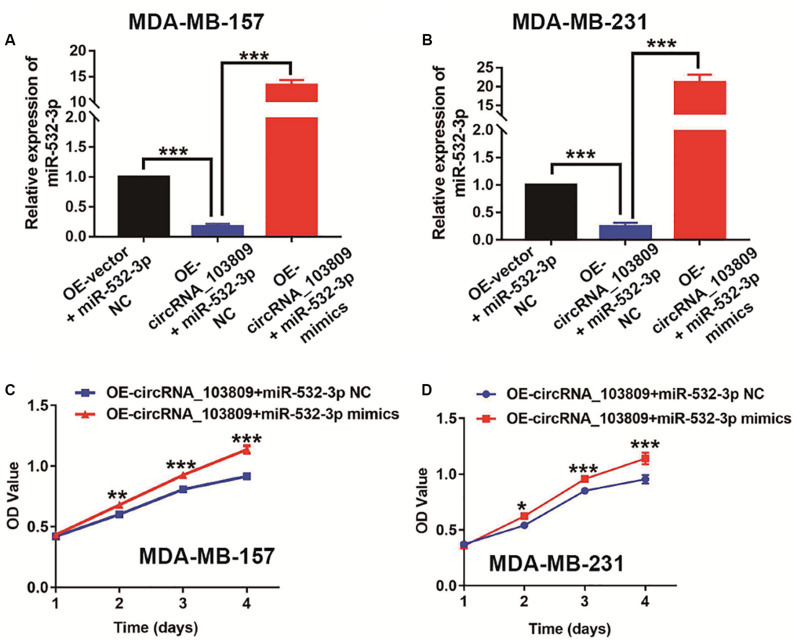
MiR-532-3p mimics reverse the antiproliferative effect induced by the overexpression of circRNA_103809 in breast cancer cells. **(A)** The expression level of miR-532-3p in circRNA_1030809 upregulated MDA-MB-157 cells and the overexpression efficiency of miR-532-3p after transfection with miR-532-3p mimics or a negative control (miR-532-3p NC) in circRNA_1030809-overexpressing MDA-MB-157 cell lines. **(B)** The expression level of miR-532-3p in circRNA_1030809 upregulated MDA-MB-231 cells and the overexpression efficiency of miR-532-3p after transfection with miR-532-3p mimics or a negative control (miR-532-3p NC) in circRNA_1030809-overexpressing MDA-MB-231 cell lines. **(C,D)** The upregulated miR-532-3p significantly inhibited the proliferation of circRNA_1030809-overexpressing breast cancer cells *in vitro* as demonstrated using the CCK-8 assay. *P < 0.05, **P < 0.01, ***P < 0.001.

Consistent with the negative relationship between circRNA_103809 and miR-532-3p, qPCR results indicated that circRNA_103809 could significantly suppress the expression of miR-532-3p in MDA-MB-157 and MDA-MB-231 cells (Figures 6A,B). Subsequently, to evaluate the relationship between miR-532-3p and cell growth, we further upregulated miR-532-3p expression in MDA-MB-157 and MDA-MB-231 cells via transfection with miR-532-3p-specific mimics. qPCR results showed that miR-532-3p expression was successfully upregulated in OE-circRNA_103809 MDA-MB-157 and MDA-MB-231 cells (Figures 6A,B). Moreover, both transfected cells were chosen for *in vitro* proliferation experiments using CCK-8. According to the results, rescue of miR-532-3p could effectively increase the proliferation of both cells (Figures 6C,D).

### MiR-532-3p Mimics Reverse the Cell Cycle Arrest Induced by the Overexpression of CircRNA_103809 in Breast Cancer

**FIGURE 7:**
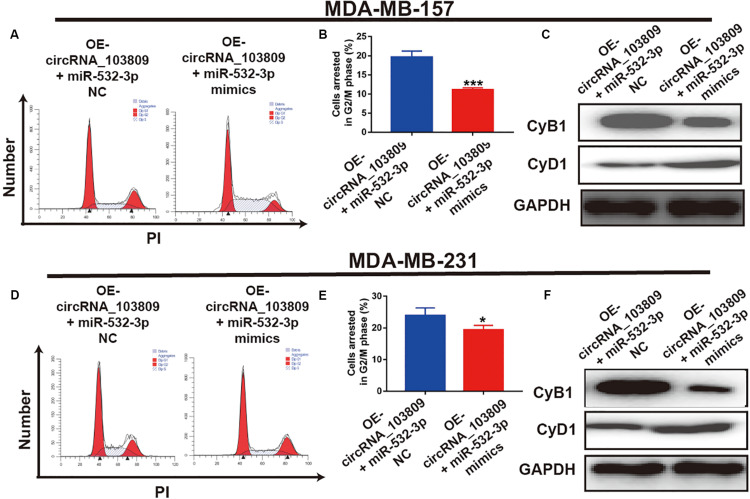
MiR-532-3p mimics reverses the cell cycle arrest induced by the overexpression of circRNA_103809 in breast cancer cells. **(A,B)** Cell cycle images (left) and flow cytometry statistical analysis (right) of circRNA_103809-overexpressing MDA-MB-157 cells after the overexpression of miR-532-3p. **(C)** The expression of cell cycle-related proteins (CyB1 and CyD1) in circRNA_103809-overexpressing MDA-MB-157 cells detected by western blot analysis after the overexpression of miR-532-3p. **(D,E)** Cell cycle images (left) and flow cytometry statistical analysis (right) of circRNA_103809-overexpressing MDA-MB-213 cells after the overexpression of miR-532-3p. **(F)** The expression of cell cycle-related proteins (CyB1 and CyD1) in circRNA_103809-overexpressing MDA-MB-213 cells detected by western blot analysis after the overexpression of miR-532-3p. All experiments were repeated at least three times. *P < 0.05, ***P < 0.001.

To further confirm the biological role of miR-532-3p in breast cancer, we detected that with the addition of miR-532-3p mimics, fewer OE-circRNA_103809 cells were arrested at the G2/M stage (Figures 7A,B). In addition, as reflected in Figure 7C, the expression of CyB1 was downregulated, while that of CyD1 protein was upregulated. Furthermore, the regulatory function of circRNA_103809 on the MDA-MB-231 cell cycle was consistently reversed with the addition of miR-532-3p (Figures 7D–F). Accordingly, the quantified western blotting bands also showed a decrease in CyB1 and an increase in CyD1 (Supplementary Material and Supplementary Figures S4A,B). These results identified that with the addition of miR-532-3p, the antitumor effects induced by the upregulation of circRNA_103809 could be reversed.

### MiR-532-3p Rescues the Inhibited Metastasis and EMT Process Induced by the Overexpression of CircRNA_103809 in Breast Cancer Cells

**FIGURE 8:**
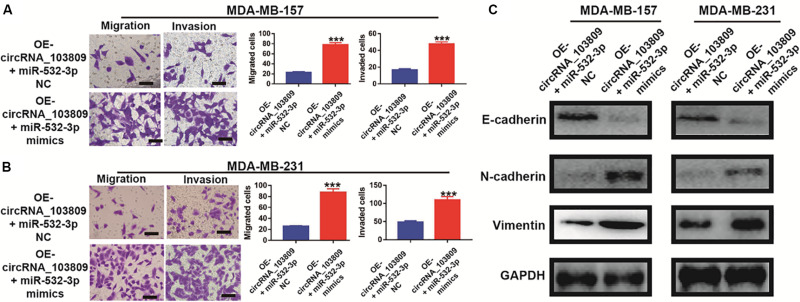
MiR-532-3p mimics rescue the inhibited metastasis and EMT process induced by the overexpression of circRNA_103809 in breast cancer cells. **(A)** Cell migration and invasion images (left) and statistical analysis (right) of migrated and invaded circRNA_103809-overexpressing MDA-MB-157 cells after the overexpression of miR-532-3p. **(B)** Cell migration and invasion images (left) and statistical analysis (right) of migrated and invaded circRNA_103809-overexpressing MDA-MB-213 cells after the overexpression of miR-532-3p. **(C)** The expression of an epithelial cell marker (E-cadherin) and mesenchymal markers (N-cadherin and vimentin) in circRNA_103809-overexpressing MDA-MB-157 and MDA-MB-213 cells detected by western blot analysis after the overexpression of miR-532-3p. All experiments were repeated at least three times. ***P < 0.001.

In this study, we discovered that with the miR-532-3p overexpression, migration and invasion were dramatically increased in OE-circRNA_103809 MDA-MB-157 cells (Figure 8A). Consistent with these results, increased migration and invasion were also observed in OE-circRNA_103809 MDA-MB-231 cells after the addition of miR-532-3p (Figure 8B). In fact, our previous studies indicated that the overexpression of circRNA_103809 could significantly increase the epithelial mark (E-ca), while mesenchymal markers including N-ca and Vimentin proteins, were consistently reduced. Therefore, we assumed that circRNA_103809 might regulate breast cancer cells’ metastasis by interpreting EMT pathway. To examine the pro-metastasis mechanism of miR-532-3p, western blotting was performed to identify the expression of EMT-related markers. After upregulating miR-532-3p expression, the epithelial mark (E-ca) was again reduced, while mesenchymal markers including N-ca and Vimentin proteins, were consistently increased in OE_ circRNA_103809 breast cancer cells, in both circRNA_103809-overexpressed MDA-MB-157 and MDA-MB-231 cells (Figure 8C). Accordingly, quantified western blotting bands also showed changes in the above three markers (Supplementary Material and Supplementary Figures S5A,B). Therefore, these results consistently revealed that circRNA_103809 could interfere the EMT pathway, then inhibit the cell metastasis via regulating miR-532-3p expression in breast cancer.

## Discussion

Breast cancer has become one of the most serious disease threatening mankind health in the world (Rahimzadeh et al., 2016; Bray et al., 2018). Thus far, surgical resection combined with chemotherapy is the most preferred therapeutic method (Curigliano et al., 2017). Furthermore, targeted chemotherapy is also an effective method for the patients with breast cancer (Logan et al., 2019; Pernas and Tolaney, 2019). For example, based on the Her-2-receptor signaling, trastuzumab antibody has been widely used in breast cancer patients with positive her-2 positive and receives good therapeutic effects (Liang et al., 2018; Montemurro et al., 2019). However, the 5-year survival rate of advanced breast cancer patients was still at a low level (Eldredge-Hindy et al., 2014). Therefore, identifying novel biomarkers and exploring their potential mechanisms is an urgent issue for the treatment of breast cancer.

Increasing studies have demonstrated that circRNAs participated in the progression of various cancers, such as gastric cancer, breast cancer and other types of tumors (Su et al., 2019; Zhang et al., 2019). For example, circular RNA AKT3 could inhibit the expression of miR-198 and up-regulate PIK3R1 expression, then enhance cisplatin resistance in gastric cancer (Huang et al., 2019). As a matter of fact, circRNA_103809 is a special non-coding RNA which is generated from the alternative splicing of the known protein-coding ZFR gene. Notably, numerous studies indicated that circRNA_103809 was involved in the development of various cancer, including lung cancer (Liu et al., 2018), colorectal cancer (Bian et al., 2018) and et al. For example, circRNA_103809 had been identified as a tumor inhibitor in liver cancer via binding to miR-620 (Li and Shen, 2019). Besides, Liu’s research demonstrated that circRNA_103809 could upregulate ZNF121-dependent MYC expression via sponging miR-4302, and finally promote cell proliferation and metastasis in lung cancer (Liu et al., 2018). However, there was still not clear whether circRNA_103809 functioned as an oncogene or tumor suppressor in breast cancer. Therefore, we performed RT-qPCR assay to detect the expression pattern of circRNA_103809 in breast cancer. In this study, our data showed that the expression of circRNA_103809 in breast cancer tissues was higher than that in adjacent normal tissues. After statistical analysis, we also discovered that the breast cancer patients with circRNA_103809 low-expression were more likely to have distant metastasis, HER-2 negative, advanced TNM stage and shorter survival rate. Taken together, circRNA_103809 might be considered as a cancer suppressor and even a prospective biomarker for breast cancer.

To further explore the biological function of circRNA_103809 in breast cancer, we constructed the circRNA_103809-overexpressed breast cancer cell lines via plasmid transfection. Subsequently, the results of CCK-8 assay showed that the overexpression of circRNA_103809 could dramatically inhibit breast cancer cell growth. In addition, with overexpression of circRNA_103809, more breast cancer cells were arrested at the G2/M phase. Furthermore, we also found that circRNA_103809 could suppress the cellular metastasis capability of breast cancer cells. Consequently, our data consistently suggested that circRNA_103809 might also act as an inhibitor in the occurrence and procession of breast cancer, but the potential regulatory mechanism remained unclear.

As we all know, epithelial-mesenchymal transition (EMT) is a biological process in which epithelioid cells transform into mesenchymal cells and then acquire enhanced migratory and invasive properties (Lee et al., 2006; Thiery et al., 2009; Brabletz et al., 2018). Increasing studies have suggested that EMT is a significant mechanism in the metastasis of malignant tumors, and this signaling pathway could be regulated by circRNAs (Fang et al., 2018; Meng et al., 2018). Furthermore, previous studies identified that circRNA_103809 could interfere with metastasis in liver cancer (Li and Shen, 2019). In our research, we also identified that the overexpression of circRNA_103809 could lead to the expression of epithelial mark (E-cadherin) increased, while the expression of mesenchymal markers (N-cadherin and vimentin) decreased in breast cancer cell lines. Therefore, we concluded that circRNA_103809 could exert an anti-metastatic effect via interfering with the EMT pathway.

In recent years, accumulating evidences have established that circRNAs could act as the miRNAs sponge to inhibit the function of the special miRNA (Panda, 2018; Yang et al., 2019). For instance, circ_0005230 could directly down-regulate the expression of miR-1238 and miR-1299, then facilitates cell growth and metastasis in cholangiocarcinoma (Xu Y. et al., 2019). Based on the results of Starbase website, we predicted miR-532-3p as a downstream target. In fact, emerging studies revealed that miR-532-3p could act as a cancer promoter or inhibitor in different kinds of cancers, such as ovarian cancer (Zhou et al., 2018), hepatocellular carcinoma (Wang et al., 2019) and et al. For example, Gu’s research revealed that miR-532-3p could suppress the colorectal cancer progression via regulating ETS1/TGM2 axis-mediated Wnt/β-catenin signaling pathway (Gu et al., 2019). Another study indicated that miR-532-3p could directly modulating CCR7 expression, following by exert antitumor effect in tongue squamous cell carcinoma (Feng et al., 2019). Furthermore, in this study, our data indicated that miR-532-3p was remarkably overexpressed in breast cancer. The results of a correlation analysis showed that miR-532-3p expression was negatively related to circRNA_103809 expression, which was also confirmed by a dual-luciferase reporter assay. Interestingly, we also found that the up-regulation of miR-532-3p could partly reverse the cell proliferation, cell cycle, migration and invasion of breast cancer cells, which was inhibited via the upregulation of circRNA_103809. Consequently, miR-532-3p might be the downstream target of circRNA_103809 in breast cancer.

## Conclusion

To sum up, this study showed that the overexpression of circRNA_103809 could directly regulated the function of miR-532-3p, followed by arrest at G2/M phase and suppression of cell proliferation and metastasis via interfering EMT signaling pathway in breast cancer. However, there are some limitations in this study. That is, the molecules downstream of the circRNA_103809/miR-532-3p axis are unknown, and *in vivo* anti-tumor effect induced by circRNA_103809 overexpression are unclear. Overall, our findings indicate that circRNA_103809 might be a promising target for breast cancer therapy.

## Data Availability Statement

All datasets generated for this study are included in the article/Supplementary Material.

## Ethics Statement

The studies involving human participants were reviewed and approved by Medical Ethics Committee of Nanfang Hospital, Southern Medical University. The patients/participants provided their written informed consent to participate in this study.

## Author Contributions

ZG and CY conceived and designed the study. ZG, ML, and JD performed the experiments. ZG and QL performed the statistical analysis. All authors wrote and revised the manuscript.

## Conflict of Interest

The authors declare that the research was conducted in the absence of any commercial or financial relationships that could be construed as a potential conflict of interest.

## Supplementary Material

FIGURE S1 The quantification of western blotting data for the protein of CyB1 and CyD1 in circRNA_103809-overexpressing MDA-MB-157 cells **(A)** and MDA-MB-231 cells **(B)**. All experiments were repeated at least three times. ^∗∗∗^*P* < 0.001.

FIGURE S2 The statistical graph of early apoptosis rate, late apoptosis rate and total apoptotic rates for the circRNA_103809-overexpressing MDA-MB-157 cells **(A)** and MDA-MB-231 cells **(B)**. All experiments were repeated at least three times.

FIGURE S3 The quantification of western blotting data for the protein of E-ca, N-ca and Vimentin in circRNA_103809-overexpressing MDA-MB-157 cells **(A)** and MDA-MB-231 cells **(B)**. All experiments were repeated at least three times. ^∗∗∗^*P* < 0.001.

FIGURE S4 The quantification of western blotting data for the protein of CyB1 and CyD1 circRNA_103809-overexpressing MDA-MB-157 cells **(A)** and MDA-MB-231 cells **(B)** after the overexpression of miR-532-3p. All experiments were repeated at least three times. ^∗∗∗^*P* < 0.001.

FIGURE S5 The quantification of western blotting data for the protein of E-ca, N-ca and Vimentin in circRNA_103809-overexpressing MDA-MB-157 cells **(A)** and MDA-MB-231 cells **(B)** after the overexpression of miR-532-3p. All experiments were repeated at least three times. ^∗∗∗^*P* < 0.001.
