# Supplementary figures and images for: CircRNA_103809 Suppresses the Proliferation and Metastasis of Breast Cancer Cells by Sponging MicroRNA-532-3p (miR-532-3p)
Source: Front Genet. 2020 May 15;11:485. doi: 10.3389/fgene.2020.00485 (PMC7243809; doi:10.3389/fgene.2020.00485)

**Supplementary files**

**Figure S1**


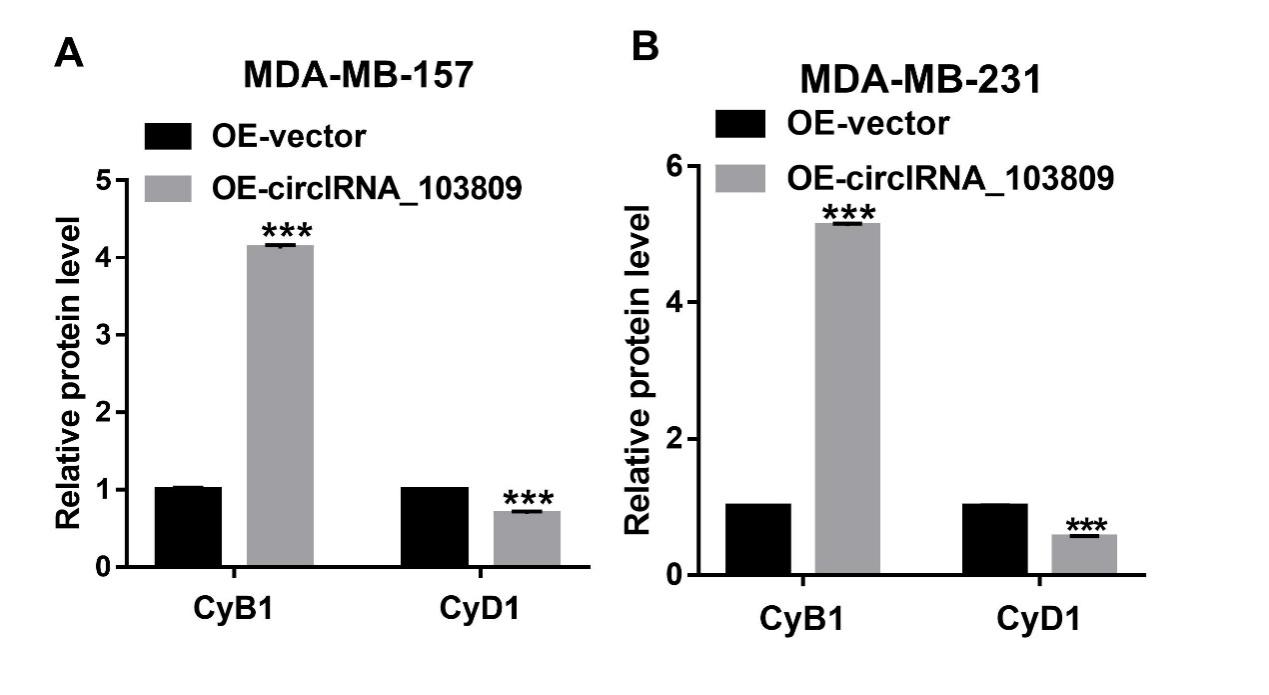


**Figure S2**


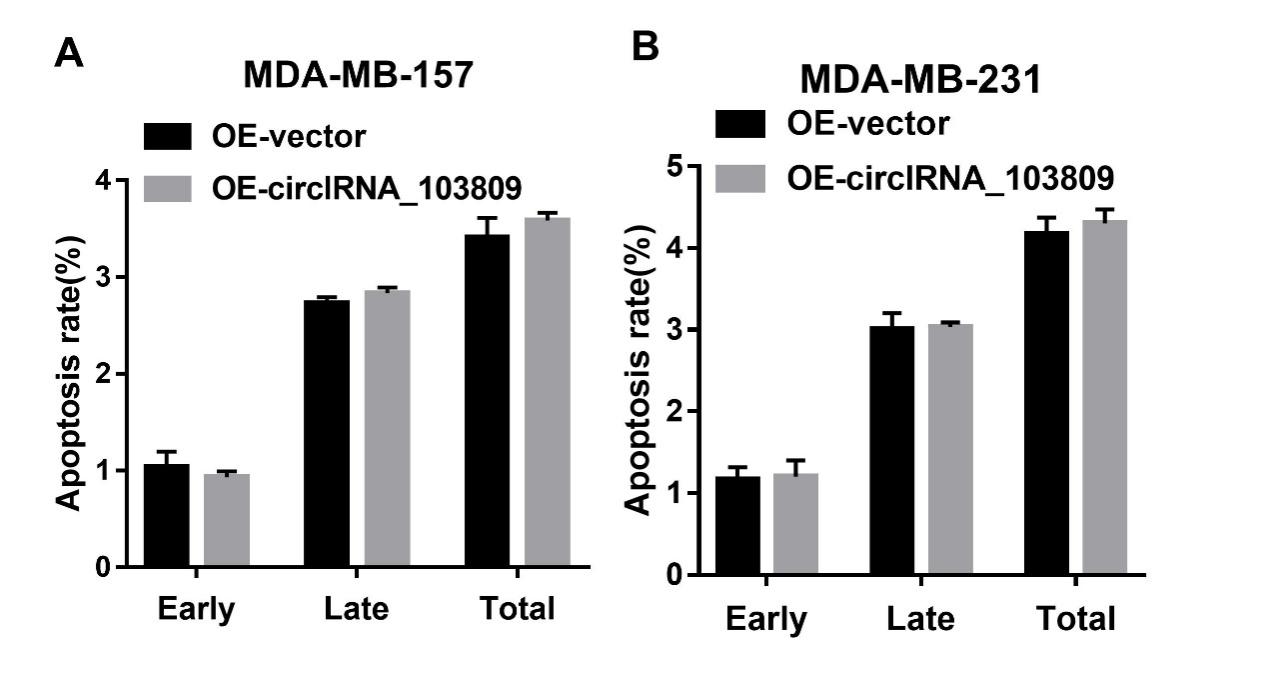


**Figure S3**


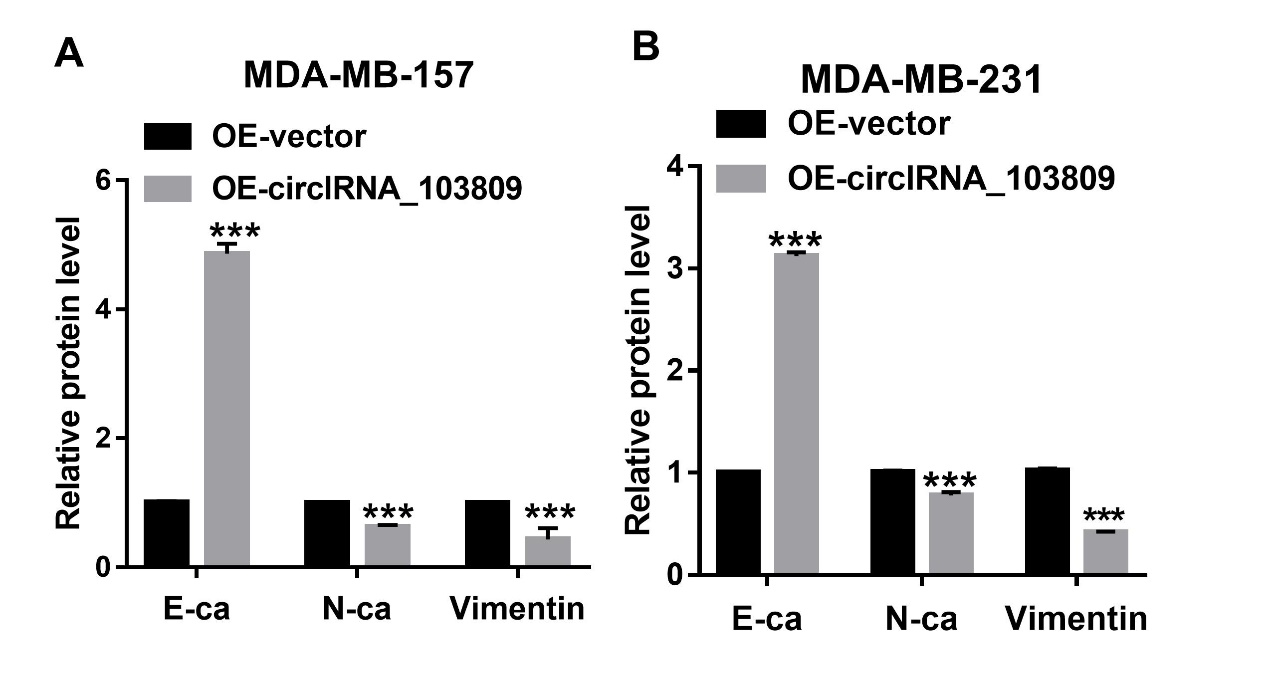


**Figure S4**

**
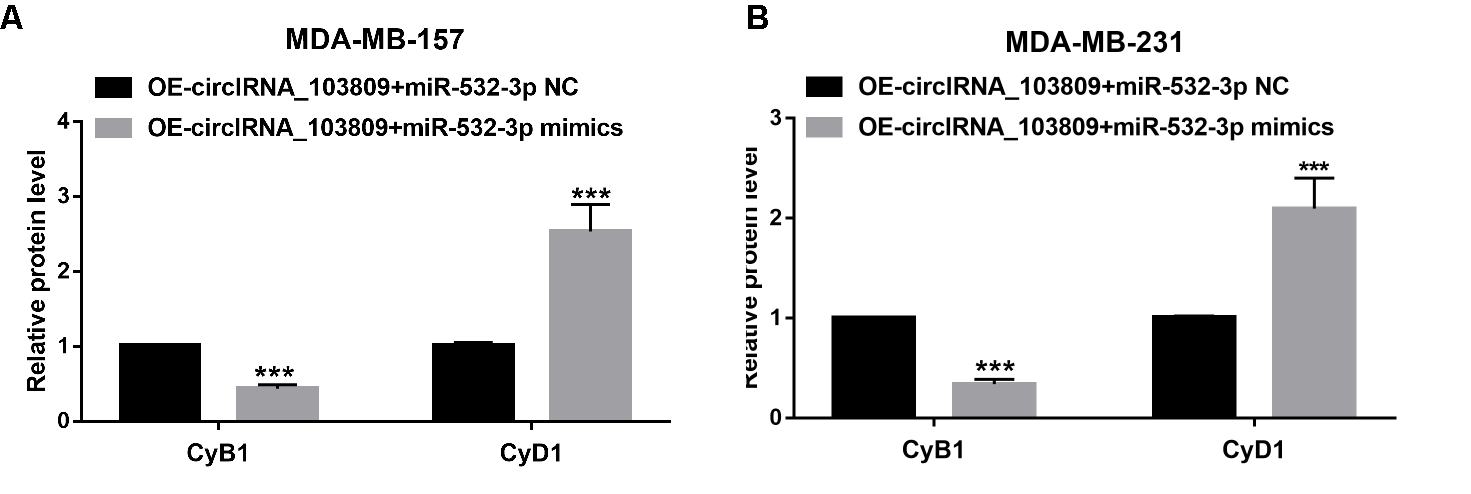
**

**Figure S5**


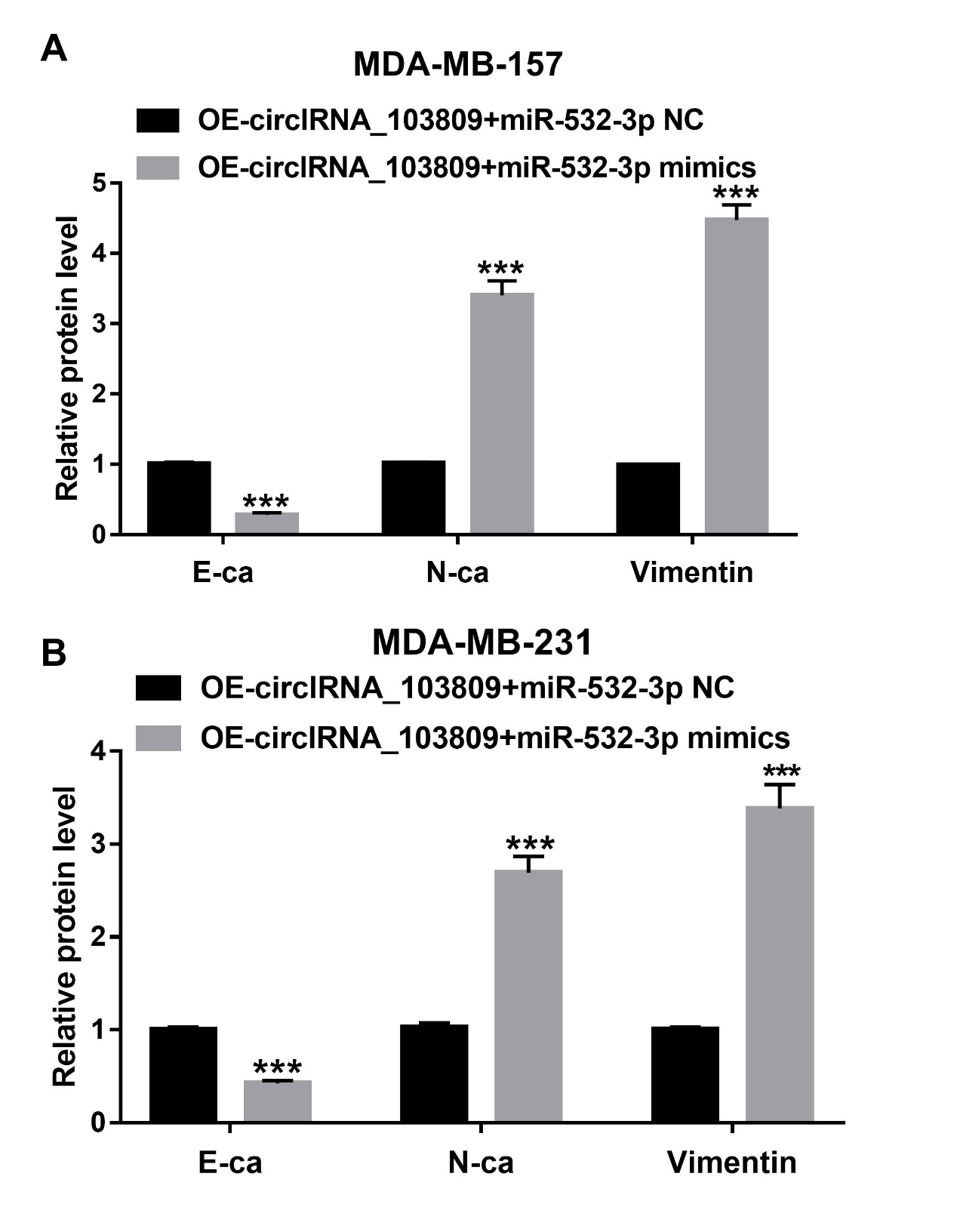

Supplement: FIGURE S1 — The quantification of western blotting data for the protein of CyB1 and CyD1 in circRNA_103809-overexpressing MDA-MB-157 cells (A) and MDA-MB-231 cells (B). All experiments were repeated at least three times. ∗∗∗P < 0.001. [file Data_Sheet_1.docx]
